# T-cell lymphoma in the nasal cavity of a Brown Swiss heifer

**DOI:** 10.1186/s13028-015-0100-8

**Published:** 2015-02-12

**Authors:** Ueli Braun, Carina Brammertz, Eva Maischberger, Danielle A Bass, Stefanie Klausmann, Titus Sydler

**Affiliations:** Department of Farm Animals, Vetsuisse Faculty, University of Zurich, CH-8057 Zurich, Switzerland; Clinic for Equine Internal Medicine, Vetsuisse Faculty, University of Zurich, CH-8057 Zurich, Switzerland; Division of Diagnostic Imaging, Vetsuisse Faculty, University of Zurich, CH-8057 Zurich, Switzerland; Institute of Veterinary Pathology, Vetsuisse Faculty, University of Zurich, CH-8057 Zurich, Switzerland

**Keywords:** Cattle, Nasal cavity, Neoplasia, T-cell lymphoma

## Abstract

**Background:**

Tumours of the upper respiratory tract are relatively common in cattle, but to our knowledge, there have been no reports of lymphoma of the nasal cavity. This case report describes the findings in a 22-month-old Brown Swiss heifer with T-cell lymphoma of the nasal cavity.

**Case presentation:**

The main clinical findings were lacrimation and swelling of the head above and below the right eye, mild exophthalmos, third eyelid prolapse, purulent ocular discharge and congestion of scleral blood vessels. An endoscope could only be introduced a few centimetres into the right nasal cavity because of an obstructing mass in the nasal passage. Radiographs showed a mass in the right nasal cavity and maxillary sinus. A tentative diagnosis of neoplasia of the right nasal cavity was made and the heifer was euthanased and necropsied. A firm, tan mass measuring 10 by 13 by 15 cm in the right half of the head occupied the entire right nasal cavity. A final diagnosis of high-grade, malignant, small-sized T-cell lymphoma was made based on histological and immunohistochemical evaluation. A distinction between αβ T-cell or γδ T-cell lymphoma was not made.

**Conclusions:**

This report on T-cell lymphoma in the nasal cavity of a cow suggests that nasal lymphoma should be included in the list of differential diagnosis of conditions associated with dyspnoea and stertorous breathing in cattle.

## Background

Diseases of the nose, conchae, ethmoid and nasopharynx are relatively common in cattle and include rhinitis, papillomatosis, actinobacillosis, nasal granuloma, foreign body, conchal cysts and tumours [1,2]. A search of the German veterinary literature from 1893 to 1973 on nasal tumours in cattle yielded 21 case reports and one publication describing 20 cases of ethmoid tumour; the latter also included single cases of anaplastic carcinoma, osteoma, osteosarcoma and osteochondroma [3]. Since then, there have been sporadic reports of bovine nasal tumours including squamous cell carcinoma [4], osteosarcoma [5], nasal chondrosarcoma [6], malignant schwannoma [7], liposarcoma [8] and osteoma [9]. We recently described a cow with dyspnoea and stertor caused by a malignant peripheral nerve sheath tumour in the nasopharynx [10]. All of the reported cattle with nasal tumours had dyspnoea and stertorous breathing sounds that originated from the nasal cavity. Sinonasal cysts must be ruled out in young cattle with these clinical signs [11]. To our knowledge, there have been no reports of lymphoma of the nasal cavity, conchae, ethmoid and nasopharynx in cattle except for one case of lymphosarcoma of the frontal sinus, which expanded into the caudodorsal part of the nasal cavity in a cow [12]. Clinical signs in that case were typical of frontal sinusitis and included extended neck, closed eyes and bilateral seromucous nasal discharge without dyspnoea or stertor. This report describes a 22-month-old Brown Swiss heifer with nasal stertor, abnormal contour of the skull in the right ocular area, exophthalmos and lacrimation related to a neoplastic mass with histological and immunohistochemical features consistent with a small-sized T-cell lymphoma.

## Case presentation

**Figure 1:**
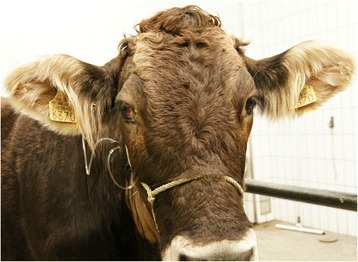
**Deformation of the head in the right periocular region.** Deformation of the head in the right periocular region in a 22-month-old Brown Swiss heifer with lymphoma of the right nasal cavity. There is swelling of the head above and below the eye, mild exophthalmus, prolapse of the third eyelid, lacrimation and traces of ocular discharge.

**Figure 2:**
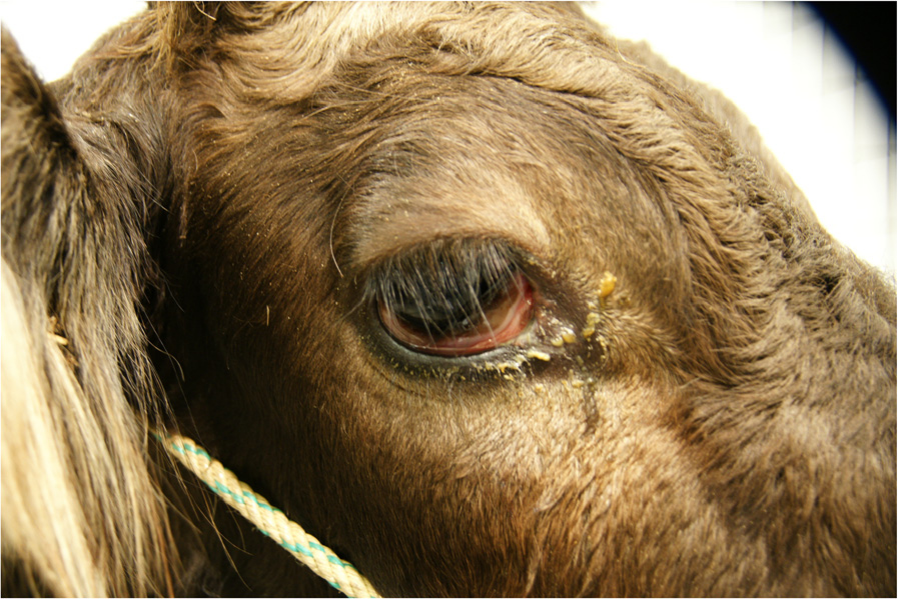
**Close**-**up of the periocular region.** Close-up of the Brown Swiss heifer with nasal lymphoma shown in Figure 1.

The heifer was referred to our clinic because of lacrimation and swelling of the right side of the head. The heifer was moderately depressed, off feed and had nasal stertor. The rectal temperature was 39.0°C, the heart rate was 66 beats per minute and the respiratory rate was 24 breaths per minute. Rumen motility was reduced, and foreign body tests and simultaneous auscultation and percussion of both sides of the abdomen were negative. The manure was olive-coloured, had a normal consistency and did not contain visible feed particles. A dipstick (Combur^9^-Test, Roche, Basel) analysis of a urine sample was normal. There was no mammary development. There was supraorbital and infraorbital swelling causing bulging of the skull around the right eye (Figures 1 and 2) and mild exophthalmus, prolapse of the third eyelid, purulent ocular discharge and congestion of scleral blood vessels. There was mild epistaxis on the right side and no movement of air from the right naris during expiration. The palpable peripheral lymph nodes including the mandibular lymph nodes were normal.

**Figure 3:**
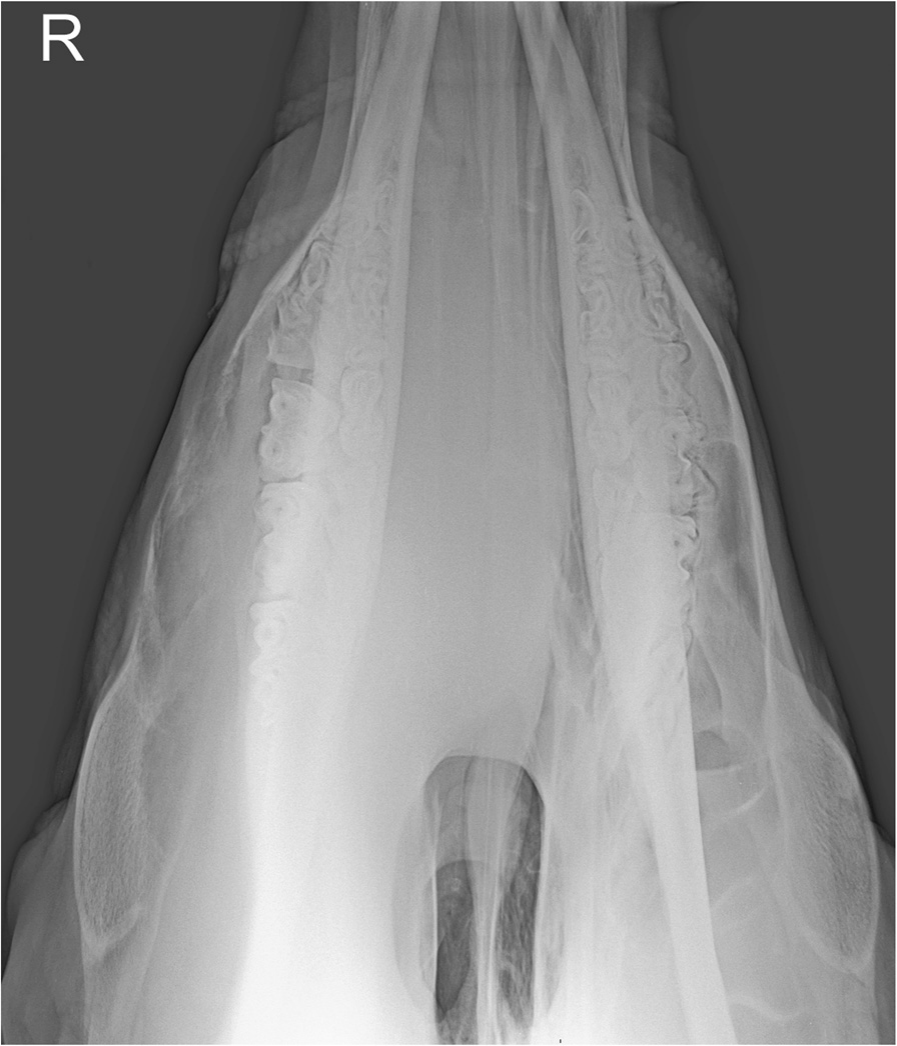
**Dorsoventral radiographic view of the head.** Dorsoventral radiographic view of the rostral part of the head of a Brown Swiss heifer with lymphoma of the right nasal cavity. A large, homogeneous, soft-tissue density mass occupies the right nasal cavity and maxillary sinus. The mass displaces the septum to the left and caused considerable thinning of the lateral wall of the right maxillary sinus.

There was mild leukocytosis (10.3 × 10^9^ leukocytes/l, normal 5–10 × 10^9^ leukocytes/l) with 50.5% segmented neutrophils, 42.0% lymphocytes, 4.0% monocytes and 3.5% eosinophils. Fibrinogen concentration was increased (8 g/l, normal 3–5 g/l) and serological testing for enzootic bovine leukosis was negative. Endoscopic examination of the left nasal cavity yielded normal findings, but examination of the right nasal cavity was impeded by a mass located a few centimetres caudal to the right nasal opening. There was a trace of blood on the ventral aspect of the right nasal opening. Radiography revealed a large space-occupying mass in the right nasal cavity and right maxillary sinus (Figure 3), and the nasal septum was slightly displaced to the left. The wall of the maxillary sinus was thinner than normal and had undergone partial osteolysis rostrally. The dental alveoli of the first and second molars were partially disintegrated. A tentative diagnosis of neoplasia of the right nasal cavity was made.

**Figure 4:**
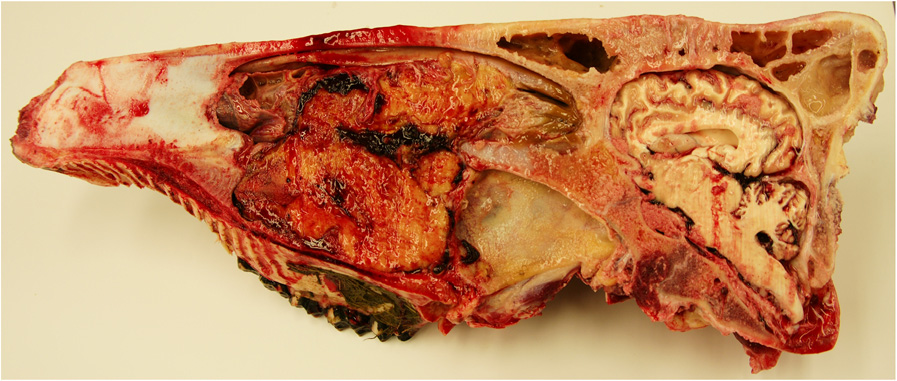
**Sagittal section of the head.** Sagittal section of the head of a Brown Swiss heifer with lymphoma of the right nasal cavity. The tumour occupies the right ventral nasal passage to the aboral end of the hard palate and has invaded the palatine sinus and the ventral concha. The hard palate is displaced ventrally.

**Figure 5:**
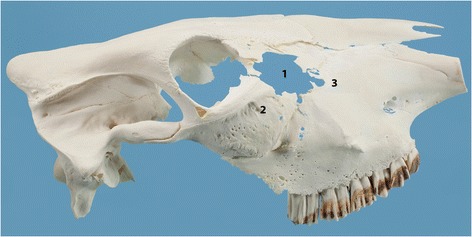
**Skull of a heifer with lymphoma of the right nasal cavity.** Skull of a 22-month-old Brown Swiss heifer with lymphoma of the right nasal cavity. There is complete osteolysis of the lacrimal bone (1), which is missing, and partial osteolysis of the dorsal part of the zygomatic bone (2) and the aboral part of the maxillary bone (3).

**Figure 6:**
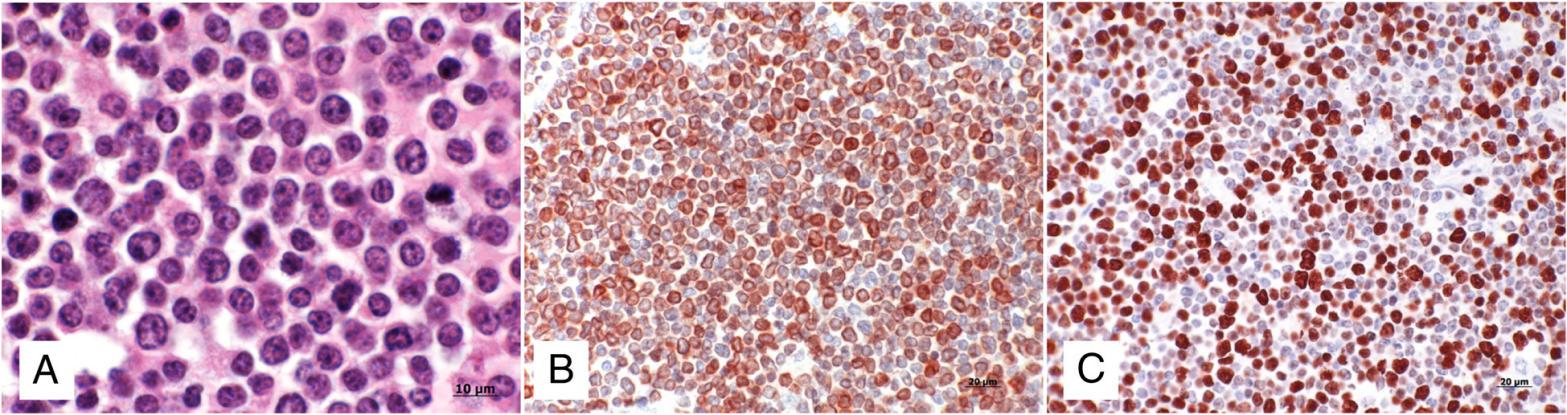
**Histological and immunohistochemical findings.** Histological and immunohistochemical findings in a Brown Swiss heifer with lymphoma of the right nasal cavity. **A)** Monomorph population of small lymphoid cells containing a round nucleus with one nucleolus and stippled chromatin surrounded by a thin rim of pale eosinophilic cytoplasm. Single cells have hyperchromatic nuclei, nuclear indentation and mild anisokaryosis. H&E staining, 100x. **B)** The majority of neoplastic cells are positive for the T-cell marker CD3. **C)** 55% of the neoplastic cells express the proliferation marker Ki-67.

**Figure 7:**
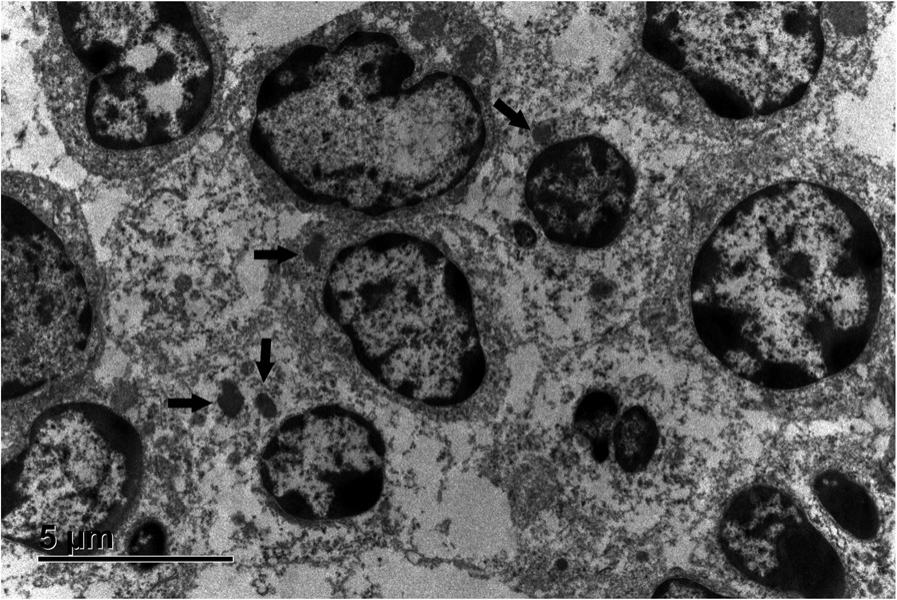
**Electron microscopic findings.** Ultrastructurally, neoplastic lymphoid cells are characterised by round or cleaved (top left) nuclei, with stippled often marginated chromatin and one nucleolus. Within the cytoplasm, single, moderately electron-dense spherical to ellipsoid granules are present (arrows). Lead citrate, uranylacetate.

The heifer was euthanased because of a poor prognosis and a postmortem examination was carried out. A firm, tan-coloured mass measuring 10 by 13 by 15 cm occupied the right ventral nasal meatus and infiltrated the ventral nasal concha (Figure 4). The mass had invaded the hard palate and the palatine and maxillary sinuses, was in contact with the right eye and protruded to the left side of the head across the midline. There was partial resorption of the hard palate and the upper jaw (Figure 5). The alveoli of the premolars and molars were exposed. The inner organs and lymph nodes were normal. Histological examination of the mass revealed densely packed round cells with an average diameter of 1.5 times the diameter of an erythrocyte. The cells had predominantly round nuclei with stippled chromatin and one nucleolus, surrounded by a thin rim of pale eosinophilic cytoplasm (Figure 6A). A few nuclei were cleaved. There was little variation in nuclear size. The mitotic index was more than 20 per 400 × field. Immunhistochemical staining was positive for CD3 in more than 50% of the neoplastic cells (Monoclonal Mouse Anti-Human CD3, Clone F7.2.38, DakoCytomation, Zug) (Figure 6B). Neoplastic cells did not stain for the B cell marker CD 79α (Monoclonal Mouse Anti-Human CD79αcy Clone HM57, DakoCytomation) or CD20 [13], however some normal CD79α and CD20 cells were stained in the unaffected parts of the mucous membranes. Further immunohistochemical characterisation of the CD3 positive neoplastic cells was not carried out because established WC1- and/or CD5-immunohistochemistry was not available. However, electron microscopy revealed intracytoplasmatic electron-dense granules in a few cells suggestive of cytotoxic granules [14,15] (Figure 7). The proliferation marker Ki-67 (Monoclonal Mouse Anti-Human Antigen Clone MIB-1, DakoCytomation) was expressed immunohistochemically by 55% of the neoplastic cells, reflecting the high proliferative rate (Figure 6C). Based on all findings, a diagnosis of sporadic bovine leukosis (SBL) in the form of atypical, primarily extranodal, highly malignant, nasal, small-sized T-cell lymphoma was made.

Nasal stertor, abnormal head contour in the area of the right eye and lacrimation were indications of a lesion in the right nasal cavity, most likely a neoplasm or sinonasal cysts. The latter are common in young cattle [11] but facial deformation is not typical. Nasal stertor is usually accompanied by inspiratory dyspnoea in cattle with sinonasal cysts. Facial deformation, mild exophthalmus and prolapse of the third eyelid were indications of a space-occupying neoplasm. Radiography of the head and endoscopic examination of the upper respiratory tract allowed localisation and visualisation of the mass. Biopsy samples were not collected because the histopathological pattern of a small tissue sample is often not representative of the main tumour mass in nasal neoplasia [16]. Computed tomography was not used because the heifer had a poor prognosis based on clinical and radiographic findings.

The main differential diagnosis of a sinonasal mass in cattle is ethmoid carcinoma [1,2,17], but the tumours previously listed must also be considered. Lymphoma was also suspected because this is the most common tumour of young cattle. Classification of bovine lymphoma is based on epidemiological and clinicotopographical criteria. The age of the patient also plays a role in classification: viral enzootic bovine leukosis occurs in adult cattle, and sporadic bovine leukosis (SBL) is seen in young cattle [18]. Enzootic bovine leukosis is endemic in the USA and occurs as multicentric lymphadenopathy in older cows, whereas SBL is the predominant type of leukosis in Europe [19]. The latter is subdivided into juvenile, thymic and skin forms, and cases that do not fit into any of these subgroups are referred to as an atypical form of SBL [19]. Grünberg and Eisenberg [19] described multicentric lymphadenopathy in adult cattle with SBL, and Hendrick [20] reported multicentric lymphadenopathy with thymic involvement and infiltration of the mandible in a feedlot heifer. Other authors used the term atypical for cases without clinical signs of lymph node enlargement [21]. No other organs, including lymph nodes, had macroscopic evidence of lymphoma in our patient. Morphological and immunophaenotypical classification of neoplastic cells is critical in the diagnosis of lymphoma [22]. Studies on the histomorphological classification of bovine lymphoma are limited [14,15,23,24]. In a large study, which used the National Cancer Institute Working Formulation to classify 1,198 cases, almost 90% of the tumours were moderate- to high-grade malignant lymphoma and approximately 20% were small cell lymphoma [23]. In our case, the average nuclear diameter of the lymphoma cells was 1.5 times the diameter of an erythrocyte, supporting the diagnosis of a small cell lymphoma [25]. CD3 immunohistochemistry was used to classify the tumor as T-cell lymphoma. Lymphoma caused by bovine leukaemia virus is B-cell lymphoma [22], whereas SBL is more likely to be associated with T cells [26]. B-cell lymphoma is also possible with SBL [22]. Subclassification of αβ T-cell or γδ T-cell lymphoma or natural killer (NK)-cell lymphoma was not done because appropriate immunohistochemistry was not available. However, bovine NK cells are CD3 negative [27]. Electron microscopy revealed intracytoplasmatic electron-dense granules in a few cells, which were suggestive of cytotoxic granules; a γδ T-cell lymphoma was therefore possible [14,15,24]. However, the tumour was not epitheliotropic and had no affinity to the nasal mucous membranes. A rim composed mainly of collagenous fibres separated the epithelial layer from the underlying lymphoma masses. Lymph nodes and other internal organs had no macroscopic evidence of tumour invasion and histological examination of both mandibular lymh nodes revealed no tumour cells. Immunohistochemical staining for CD3, CD79α and CD20 revealed regular distribution of lymph cells, and lymph node architecture was normal, pointing to an activated lymph node. The mitotic index is a good measurement *per se* for the classification of malignancy of many tumours including lymphoma. An index of greater than 20 per 400 x field indicates malignancy. Alternatively, the proportion of proliferating cells can also be estimated by immunohistochemical assay of Ki-67 antigen expression, which has proven very useful in human oncology [28]. Ki-67 is a highly conserved non-histone nuclear antigen protein involved in maintaining chromosomal stability during mitosis [29]. In dogs with highly malignant T-cell lymphoma, the Ki-67 index ranged from 50 to 70% [28]. The Ki-67 index was 55% in the present case, which was in agreement with the high mitotic index. Nasal lymphoma is very common in cats and is predominatly B-cell lymphoma [30], whereas in humans, primary nasal lymphomas is predominatly T-cell or NK/T-cell lymphoma [31].

## Conclusion

Neoplasia must be ruled out in cattle with nasal stertor accompanied by facial deformity of the periocular region. The differential diagnosis includes lymphoma even in the absence of peripheral lymph node enlargement. The CD3-positive lymphoma described in this report had a unique combination of features. It was a small-sized, highly malignant lymphoma restricted to the nasal tissues and did not involve peripheral lymph nodes or internal organs. In addition, it was not epitheliotropic but some neoplastic lymphocytes may have contained cytotoxic granules.
